# Health Complaints, Mental Status and Quality of Life among the Aquaculture Workers: A Cross-Sectional Study in Northern Region of Peninsular Malaysia

**DOI:** 10.3390/ijerph192316371

**Published:** 2022-12-06

**Authors:** Nur Syafiqah Mat Zain, Lai Kuan Lee

**Affiliations:** Food Technology Program, School of Industrial Technology, Universiti Sains Malaysia, Gelugor 11800, Pulau Pinang, Malaysia

**Keywords:** aquaculture, brackish water, freshwater, health complaints, mental status, quality of life

## Abstract

Aquaculture is seen as an essential food-producing sector for improving global food security and nutrition indices. This cross-sectional study examined the health complaints and mental health status of aquaculture workers, as well as their relationship with quality of life, with respect to the brackish water and freshwater aquaculture cultivation system in Penang, Malaysia. The workers’ health complaints were collected, and mental health status was evaluated as means of depression, anxiety, stress and self-esteem. Self-perceived quality of life was assessed using a structured questionnaire. This study involved the participation of 88 brackish water (84.6%) and 16 freshwater (15.4%) aquaculture workers. A total of 72.7% of the brackish water aquaculture workers were aged beyond 50 years old and had worked within five years (77.3%) in the aquaculture industry. Both brackish water and freshwater cultivation workers were confronted with fatigue, pain and insomnia. Up to 48%, 40.4%, 26% and 24% of them were facing depression, anxiety, stress and low self-esteem, respectively. A total of 3.4% of the brackish water aquaculture workers were having bad quality of life. The complaints of neck/shoulder/arm pain (*F* = 13.963; *p* < 0.001), back pain (*F* = 10.974; *p* < 0.01), hand/wrist pain (*F* = 8.041; *p* < 0.01), knee/hip pain (*F* = 12.910; *p* < 0.01) and insomnia (*F* = 10.936; *p* < 0.01) were correlated with bad quality of life among the workers. For mental health status, self-esteem (*F* = 4.157; *p* < 0.05) was found to be negatively correlated with quality of life scores. The results outlined the concerning level of health complaints and psychological distress among the aquaculture workers. The study emphasized the importance of developing an appropriate occupational health strategy in the aquaculture industry. Longitudinal investigations aimed to explore the effects of psychological distress on employment productivity among the high-risk workers are warranted.

## 1. Introduction

Aquaculture has been the world’s fastest-growing food production sector for the past two decades [1]. Global aquaculture production has reached 106 million tonnes in 2015, with 76.7 million tonnes contributed from aquatic animals, while the rest attributed to the aquatic plants origin [2]. This positive trend is projected to continue as the aquaculture sector plays a huge role in contributing to food security and poverty alleviation [3]. Indeed, aquaculture activity relieves some pressure on wild aquatic resources, creates jobs, enhances livelihoods along the value chains and improves human nutrition in a number of underdeveloped and developing countries [4,5,6,7,8,9]. As such, the health conditions of the aquaculture workers, either in term of physical or mental aspects, are of utmost important for the overall social welfare.

The fishery sector has been playing an important role as the major supplier of animal protein to the Malaysian population, with aquaculture farming serving as an important economic driver for the nation. In 2017, Malaysia has recorded total fishery production amounting to 1.7 million tonnes, including close to 1.5 million tonnes from capture, and 0.2 million tonnes from aquaculture (excluding seaweeds). In addition, Malaysia produced 0.2 million tonnes of farmed seaweeds as the world’s seventh largest producer, and ranked in third place for tropical carrageenan seaweed farming [10]. Collectively, the local aquaculture sector produced 391,000 tonnes of cultivated organisms, with an economic value of over USD 700 million, accounting for about 0.2 percent of Malaysia’s gross domestic product (GDP) in 2019 [11]. The growth of brackish water and freshwater culture is highly correlated with the domestic and international demands. The cumulative aquaculture production in 2020 reached 400,017 metric tonnes (mt), with a wholesale value of RM 3,114,731. The brackish water culture system contributed about 77.3 percent to the total aquaculture production, which is dominated by the cultivation of bivalve mollusks, shrimps, giant tiger prawns and marine fish species [12,13,14]. The freshwater aquaculture activities usually involve the breeding and raising of aquatic animals such as the tilapia, catfish and carp in freshwater lakes, ponds, rivers or even reservoirs for economic purposes [15].

In 2020, the aquaculture sector in Malaysia employed 0.13% of the total workforce (14.96 million people) [16]. The farming characteristic of the brackish water and freshwater aquaculture system in Malaysia is shown in Appendix A. In actual aquaculture practices, different hazards may exist due to biological differences between the brackish water and freshwater species, where the farmers must provide different care, time and pond management. Brackish water aquaculture operations are more delicate and riskier, thus necessitating greater operator attention. Particularly, shrimp aquaculture in brackish water ponds is a high-risk activity, especially for intensive production, and it necessitates stricter export compliance standards [17]. Moreover, brackish water cage-based fish farming was primarily implemented at the coastal areas, and such operations are subjected to natural conditions (climate change, weather, humidity, disasters) [18]. Production of high-value species is emphasized for selected trade markets and export activities. Whereas the freshwater aquaculture system is mainly carried out either in fish ponds, fish pens, reservoirs, ex-mining pools or on a limited scale, in rice paddies or canvas tanks. The breeding site is usually close to a freshwater river. Supply priority of aquaculture production is mainly given to local markets. Despite its wide diversification in terms of production culture systems and species, freshwater aquaculture production is relatively low compared to brackish water aquaculture [19]. In general, the aquaculture working conditions require the workers to be confronted with tasks such as feeding, cleaning, fish handling, record keeping, water quality measurements, normal fish husbandry and general cleaning of equipment and facility. In addition, daily inspection and routine job description involved the removal of dead fish, hauling the netting, cleaning nets and fish grading. Such activities involved the forceful motions of upper limbs, constrained neck postures (e.g., sorting size of fish), prolonged standing (e.g., grading), sorting and handling of heavy loads [20].

In 2019, the Department of Occupational Safety and Health (DOSH) investigated 274 occupational accidents involving workers in agriculture, fisheries and forestry in Malaysia [21]. In particular, fishing activities have particularly high occupational fatality rates, but injuries and illnesses to people working in its sub-sectors, aquaculture and fish farming, are not well understood [22]. Despite the promising aquaculture development, the aquaculture industry also poses underreported environmental threats and health risks [23,24,25]. According to Myers [26], drowning, electrocutions, falls from elevation, slips and trips, falling objects, needle stick injections, roadway collisions, strains and sprains, spine wounds, impalements, equipment overturns, dust inhalations from feed, net entanglements, boat or vehicle battery explosions and burns are among the top 15 aquaculture-specific occupational hazards as these activities necessitate specific practices [27]. Consequently, long-term exposure towards the hazards and risk resulted in chronic illness, injuries and health complaints among aquaculture workers [22].

For the past several decades, medical professionals, researchers and policy makers have been paying attention to the health implications of mental disorders [28,29]. Specifically, stress has dominated the literature as one of the most broadly researched psychosocial constructs, mainly in the work-related stress area. Work-related stress is defined as a conflict when the demands of work are high, and the worker is confronting difficulties to manage, control or cope with that stress. For aquaculture farmers in particular, physical and ergonomic exposures are very common, and workers reported psychosocial exposures including stress and a lack of control in their workday [24]. A number of studies have been undertaken to explore the influence of aquaculture farming for modulating mental health, as detailed in Table 1.

To date, approximately 149,949 workers are involved in the fisheries industry, where 20,149 culturists are engaged to the aquaculture industry in Malaysia [30]. Despite all its current recognition and relevance, several key health challenges must be addressed in order to enable sustainable aquaculture industrial growth. However, to the best of our knowledge, the study of health complaints among the workers in brackish water and freshwater aquacultures are comparatively under-researched. Additionally, there have been no scoping studies to examine the mental health status among the aquaculture farming communities in Malaysia. With the aforementioned, this research is aimed to assess the health complaints, mental health status and quality of life (QoL) of aquaculture workers (both brackish water and freshwater) in Penang, the top aquaculture producer in the northern region of Peninsular Malaysia. The study also evaluated the relationship between health complaints, mental health status and QoL among the aquaculture workers.

**Table 1:** Relationship between aquaculture activity and mental health status.

Ref	Country	Aquaculture System	Major Outcomes
[22]	Finland	Not mentioned	Physical or mental stress up to 19.2%
[31]	Australia	Not mentioned	Mental diseases (1.7%) Absence of anxiety/stress disorder (1.7%)
[32]	United Kingdom	Freshwater (Tilapia)	Absence of noise, smell and heat stress Discomfort, stress and infections (mental disease) Absence of feed administration, dust irritation and muscle strain (mental and physical) Absence of lighting eye strain, poor concentration and stress (mental, physical)
[33]	Netherland	Fishing villages	Psychological distress
[34]	Taiwan	Not mentioned	Fishery workers were more impacted by cardiometabolic diseases, mental illness, infection and malignancy
Current study	Malaysia	Brackish water and Freshwater	Depression (48%) Anxiety (40.4%) Stress (26%) Low self-esteem (24%)

## 2. Materials and Methods

### 2.1. Study Area

Penang is located at the northwestern coast of Peninsular Malaysia. This state is divided into two parts: the Penang Island and the mainland of Seberang Perai. In this study, aquaculture farms located at the five administrative districts, namely the North-East, South-West, North Seberang Perai, Central Seberang Perai and South Seberang Perai districts were invited to participate in this study. Penang alone has captured more than half of the market share in the RM 3 billion aquaculture industry, and produced 47,742 metric tonnes of fishery products worth RM 1.67 billion in 2018, more than half of the country’s overall total [35]. The state is currently the second largest producer of aquaculture products in the country after Sabah. Hence, Penang could serve as a platform to represent the aquaculture industry in Malaysia [35].

Penang’s aquaculture production gained the highest wholesale revenue in Malaysia. Brackish water ponds and cages constitute the majority of Penang’s aquaculture and reported the highest number of culturists. Fisheries from brackish water have been contributing nearly 50% of the total fish production, and about 69% of its value in Penang [35]. Of these, sea bass and snapper recorded the highest production, followed by shrimp, cockle and other brackish water cages species, such as hybrid grouper and mackerel.

### 2.2. Study Design

This is a cross-sectional study. Ethical approval for the research was granted from the Human Research Ethics Committee of Universiti Sains Malaysia (USM) prior to the commencement of the study (Approval code: USM/JEPeM/20120635).

### 2.3. Sampling Method and Subject Recruitment

The research team obtained a list of registered aquaculture farms (as of mid-2021) for both brackish water (n = 328) and freshwater aquaculture (n = 102) from the Penang State Fisheries Department. Subsequently, an invitation letter was issued officially to all the listed aquaculture stakeholders, followed by individual telephone calls and research intention briefing. The aquaculture stakeholders were informed with regards to the objectives and research methodologies. Reluctant participation was recorded, and those who agreed to participate were appointed with preferred dates of actual farm site visits and interviews. During the actual interview session, one aquaculture worker represented the respective farm and underwent the interview process during the working time. The interviewees were interviewed without the presence of their aquaculture stakeholder. In general, one out of five employees from each farm was involved in the interview. Data sampling was conducted from February 2021 to July 2021 by a trained enumerator.

**Figure 1:**
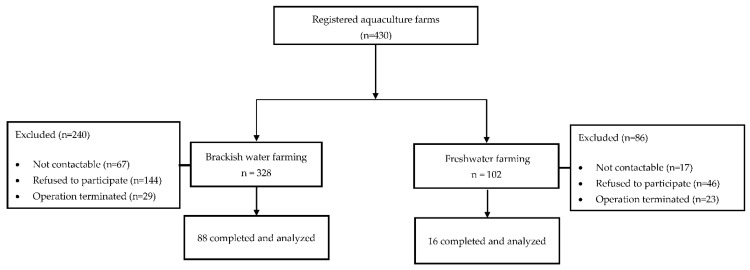
Subject recruitment.

The inclusion criteria was aquaculture workers from all registered aquaculture farms in Pulau Pinang (n = 430). For brackish water (n = 328) aquaculture farms, 67, 144 and 29 farms were excluded due to not contactable, refused to participate and terminated operation, respectively. Similarly, 17, 46 and 23 aquaculture farms were excluded from the freshwater cultivation system (n = 102) due to not contactable, refused to participate and terminated operation, respectively (Figure 1). Hence, data collection only involved workers from 88 brackish water and 16 freshwater aquaculture farms.

### 2.4. Research Instrument

A structured questionnaire has been used to access the socio-demographic background, health complaints, mental status and quality of life of the aquaculture workers.

#### 2.4.1. Socio-Demographic and Working Background

This involved the gathering of socio-demographics characteristics, such as the gender, age, household size and wealth group. Information about the fish production and aquaculture sites was sought and recorded as necessary.

#### 2.4.2. Assessment of Health Complaints

Participants graded their health complaints (health issues in general) based on a five-point Likert scale: (1) strongly disagree, (2) disagree, (3) neutral, (4) agree and (5) strongly agree. Respondents who answered agree or strongly agree were considered to experience health complaints. Aquaculture workers were asked to report the presence of the health complaints: neck/shoulder/arm pain, back pain, hand/wrist pain, knee/hip pain, fatigue, insomnia, skin ailments, gastrointestinal discomfort, respiratory problems, cardiovascular disease, white finger and allergy at the current state. The scorings were summed and classified into two categories, the “0 = no complaint” or “1 = with complaint”.

#### 2.4.3. Mental Health Assessments

(1) Depression, Anxiety and Stress Scale-42 (DASS-42)

The DASS-42 is a set of self-measures for three negative emotional states: depression, anxiety and stress, with fourteen questions for each of these three subscales [36]. The characteristic of dysphoria, hopelessness, devaluation of life, self-deprecation, interest/involvement, anhedonia and inertia were assessed. In comparison, the anxiety scale evaluates autonomic arousal, skeletal muscle, situational anxiety and subjective experience of anxious affect. The stress scale is used to examine the levels of chronic non-specific arousal in terms of difficulty in relaxation, nervous arousal, and being easily upset/agitated, irritable/over-reactive and impatient. The aquaculture workers rated the degree to which they experienced each state in the previous week. A four-point severity/frequency scale was used, and the extent of severity was indicated by a standard severity rating index.

The cut-off point adapted from previous study [37] was used to determine the respondents’ level of mental health. The cut-off score for depression was 0–9 (normal), 10–13 (mild), 14–20 (moderate), 21–27 (high) and >28 (very high). The cut-off points for anxiety were 0–7 (normal), 8–9 (mild), 10–14 (moderate), 15–19 (high) and >20 (very high). The cut-off points for stress were 0–14 (normal), 15–18 (mild), 19–25 (moderate), 26–33 (high) and >34 (very high) [38,39].

(2) The Rosenberg Self-Esteem Scale (RSES)

The Rosenberg self-esteem scale (RSES) is a self-administered scale for the estimation of overall worthiness [40,41,42]. The summed scores of 10 items were calculated, and those who scored under 15 were indicated as having low self-esteem.

#### 2.4.4. Assessment of Quality of Life

The twelve-item general health questionnaire (GHQ-12) is derived from the Goldberg general health questionnaire, which reflected respondents’ mental health status through twelve-item self-assessment results [43]. GHQ-12 has been widely used in both the clinical setting and general population as self-assessment tool. The GHQ-12 is comprised of six positive items (e.g., “Been able to enjoy your normal day-to-day activities”) and six negative items (e.g., “Been thinking of yourself as a worthless person”). Each item assesses the severity of a mental problem and the scale points are described as follows: “less than usual (0)”, “no more than usual (1)”, “rather more than usual (2)” and “much more than usual (3)”. Based on the response, the overall twelve individual items were scored as 0, 0, 1 or 1, respectively. The scores were summed to give an overall GHQ-12 scale running from 0 (the least distressed) to 12 (the most distressed). The scores ranging between 0–4 were categorized into the dichotomous scale “0 = good quality of life”, while a summed score ranging between 5 to 12 was classified as “1 = have bad quality of life”.

### 2.5. Statistical Analysis

The normality of data was analyzed using the Shapiro–Wilk test, with a significance value of *p* > 0.05 indicating normal distribution. Three types of data analysis were adopted in this study. Firstly, categorical variables were analyzed using frequency distribution. The differences in mean scores of depression, anxiety, stress and self-esteem, and QoL were tested using Independent Student’s *t* test. Simple linear regression analysis (method: enter) was used to assess the relationship between (i) health complaints and QoL, and (ii) mental health and QoL. The statistical analysis was conducted using the SPSS software version 27.0 (SPSS, Chicago, IL, USA), with the significance level defined as *p* < 0.05.

## 3. Results

**Figure 2:**
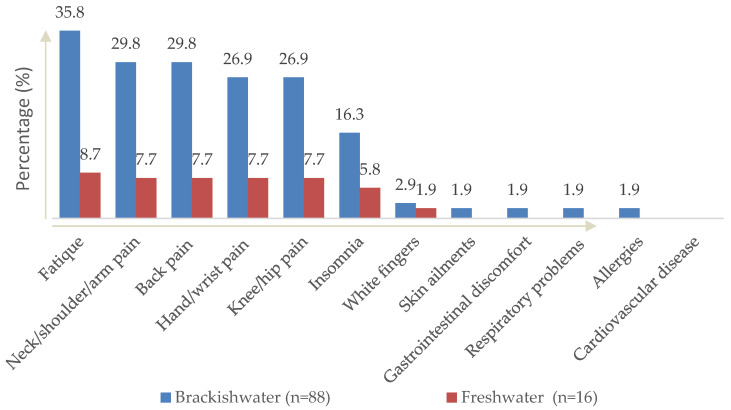
Health complaints among the brackish water and freshwater aquaculture workers.

The current study assessed the health complaints, mental status and QoL status of brackish water and freshwater aquaculture workers. The socio-demographic status of the participants is presented (Table 2), followed by the results presentation of health complaints (Figure 2), mental status (Table 3) and QoL (Table 4). The relationship between health complaints, mental health and QoL were studied (Table 5).

### 3.1. Socio-Demographic Characteristics

Table 2 shows the socio-demographic characteristics comparing the brackish water and freshwater aquaculture workers. The current study involved the participation of 84.6% of brackish water workers, with sea bass (*Lates calcarifer*), mangrove snapper (*Lutjanus argentimaculatus*) and red snapper (*Lutjanus erythropterus*) recorded as the top commodity values cultured species. The remaining 15.4% of the freshwater aquaculture workers dominated the cultivation of catfish (*Clarias* spp.), climbing perch (*Anabas testudineus*) and red tilapia (*Oreochromis* spp.). Predominantly, the brackish water workers were aged 50 years and above (72.7%), while 62.5% of the freshwater aquaculture farms employed younger workers. Majority of the brackish water (86.4%) and freshwater (75.0%) aquaculture workers belonged to the M40 and T20 socioeconomic groups. Up to 64.8% of the brackish water aquaculture reported the usage of non-fish ponds (fiber tank, floating cage), while three-quarters of the freshwater aquaculture utilized fish ponds. In term of fish productions, brackish water (79.5%) and freshwater (100.0%) cultivation produced ≤20 tonnes of aquaculture yield per each completed cycle.

### 3.2. Health Complaints Status

Figure 2 shows the health complaints reported by the aquaculture workers according to the types of cultivation. The health complaints for brackish water aquaculture in descending order were found to be being fatigue (35.8%), followed by neck/shoulder/arm pain (29.8%), back pain (29.8%), hand/wrist pain (26.9%), knee/hip pain (26.9%), insomnia (16.1%), white fingers (2.9%), skin ailments (1.9%), gastrointestinal discomfort (1.9%), respiratory problems (1.9%) and allergy (1.9%), respectively. Meanwhile, the most common types of health complaints reported by the freshwater aquaculture workers were fatigue (8.7%), followed by neck/shoulder/arm pain (7.7%), back pain (7.7%), hand/wrist pain (7.7%), knee/hip pain (7.7%), insomnia (5.8%) and white fingers (1.9%).

### 3.3. Mental Health Status

Table 3 reports the mental health status, as indicated by the classifications of depression, stress and anxiety conditions among the aquaculture workers. Up to 48%, 40.4% and 26% of the aquaculture workers were facing depression, anxiety and stress, respectively. Meanwhile, 24% of the aquaculture workers were confronted with low self-esteem.

### 3.4. Quality of Life Assessment

Table 4 shows the QoL status, as indicated by the classifications of either good or bad QoL. A total of 3.4% (n = 3) of the brackish water aquaculture workers were having bad QoL. Surprisingly, for freshwater aquaculture workers, none of them perceived bad QoL.

### 3.5. Relationship between Health Complaints, Mental Health and QoL

Table 5 reports the relationship between health complaints, mental health and QoL among the aquaculture workers. Significant positive correlations were observed between the complaints of neck/shoulder/arm pain (*F* = 13.963; *p* < 0.0001), back pain (*F* = 10.974; *p* = 0.001), hand/wrist pain (*F* = 8.041; *p* = 0.006), knee/hip pain (*F* = 12.910; *p* = 0.001) and insomnia (*F* = 10.936; *p* = 0.001) with bad QoL. For mental health status, self-esteem (*F* = 4.157; *p* = 0.044) was found to be negatively correlated with QoL scores.

## 4. Discussion

To the best of our knowledge, this is the first community study to investigate the health complaints, mental status and QoL among the brackish water and freshwater aquaculture workers in Malaysia. The survey included general questions about the workers characteristics (gender, age, year of service, household size, wealth group, types of facilities, fish production and aquaculture sites), as well as investigations into the perspectives of health complaints, mental status and QoL of aquaculture workers in Penang. One typical characteristic of the current brackish water aquaculture communities was that about three-quarters of them had less than 5 years of working experience. This could be attributed to the challenging nature of the working condition in the brackish aquaculture farms or the typical breeding fish species. The challenging working conditions in brackish water farming includes the unpredictable climate change and comparably high occupational risk during daily task implementation. Long-term exposure to challenging working conditions may result in health issues that lead to quitting the job. In addition, brackish water fish breeding is usually confronted with salinity fluctuation, maintenance of position and bacterial infection.

An important finding was that the brackish water aquaculture workers were confronted with pronounced health complaints, especially comorbidities related to pain and insomnia. Contradictorily to an earlier study from India, Borah [44] reported that the ergonomic hazards were prevalent among the freshwater aquaculture workers. We assumed that the environment threat is commonly encountered in brackish water cultivation, where the majority of the physical tasks were risky and involved manual operation. Farmers who worked long hours and performed heavy labor tasks had little time to rest, subsequently leading to sleeping difficulties [45]. This prolonged sequence of physical activities may lead to prolonged fatigue. Fatigue is presumed to be a contributing factor in farm-related workplace accidents that result in severe injury or death [46].

The present work also demonstrated that the symptoms of elevated musculoskeletal discomfort were reported in over one-third of the studied population. The results were in line with the study conducted by Mitchell and Lystad [31] in Australia, where musculoskeletal disorders (MSDs) accounted for more than one-third of overall claims, probably caused by handling, lifting or carrying heavy loads in an extended period of time. Furthermore, squatting postures in a long-term manner added excessive strain on the knee and joints [47]. All work-related disease naturally progresses to worsen over time and may eventually lead to permanent disability without early treatment or intervention [44]. In Taiwan, Chen [48] reported that a large portion of the aquaculture workers exhibited a high prevalence of cardiovascular disease, which was undetectable in the current study. However, it could be that majority of the participants were oblivious to their illness or disability [49]. Such prediction warrants future research, as it could be that the aquaculture workers in the current study lived healthier and thus reported less cardiovascular issues.

We found significant relationships between neck/shoulder/arm pain, back pain, hand/wrist pain, knee/hip pain and insomnia with QoL. Indeed, MSDs were found to be directly related to QoL by driving a negative impact on daily life function, psychological health, work capacity and income [50,51]. In addition, physically strenuous work is also associated with sleep-related problems [52,53]. Healthy sleep is fundamental to human health, as workers who suffer from insomnia are far more likely to be involved in a work-related accident than those who do not suffer from sleep disorders [54,55]. Promotion of sleep health education, as well as attention to more ergonomic working methods are important steps in improving health and promoting safety in the workplace.

The current research showed that only self-esteem was likely to have an inverse relationship with QoL. Previous findings highlighted the detrimental effects of physical and mental problems towards overall well-being in aquaculture workers [22,31,33,34], particularly psychological distress and mental illness. Research suggested that implementing psychiatric interventions could improve self-esteem [56]. Perhaps most imperative is the development of focused interventions such as mental health literacy, which addresses specific social, environmental and cultural factors affecting mental health. In parallel, prioritizing potential stakeholders with the aim to collect resourceful information about their workers, evaluation of their role as key support system and reviewing the future use in mental health policies are needed for efficacy measures.

Overall, depression, anxiety and stress levels in the aquaculture industry were moderate, but if many dangerous jobs are performed during working hours, it is likely to have a long-term negative impact on mental health, resulting in lower work productivity. Workers with depression and anxiety could have occupational role dysfunction and stress at the workplace, leading to an unhealthy working environment. In addition, the effects of anxiety, depression and psychological stress on aquaculture productivity have not been well studied; however, anxiety and depression have been shown to increase both absenteeism and presenteeism (working while sick), and presenteeism has been linked to work deterioration and productivity loss [57,58]. A mentally healthy workplace has high productivity levels, is efficient and is open to discussions about mental health issues [59].

The present work has several strengths. Firstly, this is an early study to investigate the mental health status among brackish water and freshwater workers in Penang, an economically valued northern state in Peninsular Malaysia. The presence of health complaints was subsequently captured, thereby bridging the research gap in the pool of currently available data. We highlighted limitations observed in this study. Although health complaints in the brackish water aquaculture were much higher than the freshwater aquaculture; however, it did not specify the main cause of work-related sickness. More in-depth research into the precise tasks performed at the aquaculture farm is recommended. It would be beneficial to study the risk factors associated with health complaints. The influence of genetic background, psychosocial factors and extensive study of different types of occupational tasks are fundamental to reveal the occurrence of health complaints. Thirdly, the participation was solely based on voluntary basis. Despite efforts which have been implemented to ensure optimum participation, the response rate was considered low for both brackish water and freshwater aquaculture stakeholders. Reluctant participations must be figure out and reasoned, and future research that involves a larger sample size could further improve and verify these findings.

This study is important to inform the aquaculture stakeholders and policy makers that continuous planning and strategies are warranted to review the health complaints and mental health issues in this economically important sector. As pain complaints seem consistent for both the brackish and freshwater cultivation systems, pain management should be in line and cultivated at the earliest convenience. Working condition improvement is feasible in terms of shift reschedules, encouraging task breaks, cultivating good coworker and supervisor relationships and establishing policies and systems aimed at improving QoL and promoting workplace safety [60]. Medical aid and medical coverage should be monitored and responsible to working-related injuries and diseases. The success of behavioral and mental health interventions is typically vital to alleviate psychological distress among the aquaculture workers.

## 5. Conclusions

The study identified potential health concerns among the aquaculture workers, and the findings reinforced the compelling need for preventive strategies and tackling measures. A large proportion of the aquaculture workers expressed health complaints and mental health problems, and these were positively correlated with quality of life. There is a need to investigate the differences regarding these two types of farming using larger and comparable samples. Furthermore, the underlying public health issues require full attention from the policy makers, aquaculture stakeholders and front line workers in order to achieve a sustainable aquaculture industry. A pressing need to address such occupational exposures and risks is therefore vital, and the assessment, communication, mitigation, protection from and prevention of hazards should be systematically pursued and prioritized.

## Figures and Tables

**Table 2:** Baseline characteristics of brackish water and freshwater aquaculture workers.

Characteristics	Brackish Water (n = 88)	Freshwater (n = 16)
Gender		
Male	88 (100.0)	16 (100.0)
Years of service		
≤5 years	68 (77.3)	6 (37.5)
>6 years	20 (22.7)	10 (62.5)
Age		
21–50	24 (27.3)	10 (62.5)
>50	64 (72.7)	6 (37.5)
Household size		
1–5	83 (94.3)	13 (81.3)
>6	5 (5.7)	3 (18.7)
Household income		
B40	12 (13.6)	4 (25.0)
M40 and T20	76 (86.4)	12 (75.0)
Types of facilities		
Fish pond	31 (35.2)	12 (75.0)
Non-fish pond	57 (64.8)	4 (25.0)
Fish production per cycle		
≤20 tonnes	70 (79.5)	16 (100.0)
>21 tonnes	18 (20.5)	0 (0)
Aquaculture farms		
North-East district	10 (11.4)	1 (6.3)
South-West district	6 (6.8)	4 (25.0)
North Seberang Perai	0 (0.0)	9 (56.1)
Central Seberang Perai	0 (0.0)	1 (6.3)
South Seberang Perai	72 (81.8)	1 (6.3)

Data is shown as N (%). Fish production per completed cycle was referring to individual aquaculture farming. Household income classification (B40 = <1027 USD; M40 = 1027–2322 USD; T20 = >2322 USD per month).

**Table 3:** Mental health status among the aquaculture workers.

Parameters	Normal	Mild	Moderate	High	Very High
Depression	54 (52%)	33 (31.7%)	17 (16.3%)	0	0
Anxiety	62 (59.6%)	24 (23.1%)	18 (17.3%)	0	0
Stress	77 (74%)	20 (19.3%)	7 (6.7%)	0	0
Self-esteem	79 (76.0%)	25 (24.0%)	0	0	0

Data are reported as n (%).

**Table 4:** QoL status among the aquaculture workers.

Cultivation	Mean (sd)	Good QoL (0–4)	Bad QoL (5–12)
Brackish water (n = 88)	1.58 (1.41)	85 (96.6%)	3 (3.4%)
Freshwater (n = 16)	0.00 (0.00)	16 (100.0%)	0 (0.0%)

Data for QoL was presented as n (%).

**Table 5:** Relationship between health complaints, mental health and QoL scores.

	Quality of Life				
	*F*	*β*	𝑅	*r* ^2^	*p*
**Health Complaints**					
Neck/shoulder/arm pain	13.963	0.399	0.347	0.120	<0.0001 ***
Back pain	10.974	0.344	0.312	0.097	0.001 **
Hand/wrist pain	8.041	0.299	0.270	0.073	0.006 **
Knee/hip pain	12.910	0.397	0.335	0.112	0.001 **
Fatigue	1.393	0.119	0.116	0.013	0.241
Insomnia	10.936	0.427	0.311	0.097	0.001 **
Skin ailments	0.009	−0.022	0.009	0.000	0.926
Gastrointestinal discomfort	0.356	0.139	0.059	0.003	0.552
Respiratory problems	1.045	−0.239	0.101	0.010	0.309
White fingers	2.971	−0.282	0.168	0.028	0.088
Allergies	1.045	−0.239	0.101	0.010	0.309
**Mental Health**					
Depression	1.258	−0.036	0.110	0.012	0.265
Anxiety	0.196	−0.021	0.044	0.002	0.659
Stress	0.006	−0.003	0.008	0.000	0.937
Self-esteem	4.157	−0.194	0.198	0.390	0.044 *

* *p* < 0.05. ** *p* < 0.01. *** *p* < 0.0001.

## Data Availability

Data are available from the corresponding author on reasonable request.

## Appendix A

**Table A1:** Farming characteristics of the brackish water and freshwater aquaculture system in Malaysia.

Characteristic	Brackish Water Cultivation	Freshwater Cultivation
Human resources	7%: brackish water and floating net-cage culture in the lagoons and coastal waters 6%: brackish water pond culture systems for black tiger shrimp and marine fish hatcheries 4%: bivalve mollusk culture 3%: seaweeds cultivation	70%: freshwater pond and concrete tank culture system 10%: floating net-cage culture in lakes, reservoirs, ex-mining pools and freshwater lagoons
Cultured species	Seaweeds, white leg shrimps, sea bass, tiger shrimp, cockles, grouper, red snapper, mangrove snapper, horse mackerel, milkfish, oysters, mussels and others	Red tilapia, freshwater catfish, river catfish, Labeo rohita, black tilapia, bighead carp, river carp, common carp, Javanese carp, grass carp, giant freshwater prawn, snakehead, giant snakehead and others
Most profitable species	White leg shrimps	Red tilapia
Production	144,189 tonnes	49,951 tonnes
Size	17,357 ha	4769 ha
Practices/systems	Ponds, cages and raft systems for mussel and oyster Bottom culture for cockle Long line for seaweed	Ponds, used-mining pools, tanks, cages and pen culture systems
Market and trade	Singapore, Taiwan, China and Hong Kong: barramundi, groupers, crabs, black tiger prawns, white leg shrimps Europe Union, Japan, United States of America and Australia: black tiger prawns and white leg shrimp are exported as block frozen or as value added products	Domestic consumption
Company operation years	6 to 10 years	≤5 years
Number of employees	20,262	15,719
Company size (no. of workers)	1–10	1–5
Income (per worker per month)	≥423.50 USD	≤317.63 USD

Sources: [12,17,61].
